# Syndemics of Severity and Frequency of Elder Abuse: A Cross-Sectional Study in Mexican Older Females

**DOI:** 10.3389/fpsyt.2018.00599

**Published:** 2018-12-18

**Authors:** Mireya Vilar-Compte, Pablo Gaitán-Rossi

**Affiliations:** EQUIDE Research Institute for Equitable Development, Universidad Iberoamericana, Mexico City, Mexico

**Keywords:** elder abuse syndemics, elder abuse severtity, social determinants, depression and elder abuse, elder abuse in Mexico

## Abstract

**Background:** Elder abuse is a common phenomenon with important effects on the health and well-being of older adults. There are important gaps in elder abuse measurement, as it is usually reported as the absence or presence of elder abuse, disregarding its severity and frequency.

**Objectives:** Identify different ways of measuring severity and frequency of elder abuse and assess whether different experiences of severity and frequency suggest syndemic relationships.

**Methods:** Through a sample of 534 non-institutionalized Mexican older women, we assessed how severity (i.e., number of abusive experiences and number of types of abuses) and frequency (i.e., if abusive experiences had happened few or many times) correlate among them. For each of these measures we estimated a multinomial model to examine associations with social support, functional impairments, socioeconomic status, food insecurity, depression, and comorbidities, while controlling for key socio-demographic variables.

**Results:** 30.5% of the older women reported psychological abuse, 8.2% financial exploitation, 5.1% caregiver neglect, 3.5% physical abuse, and 1.2% sexual abuse. In terms of frequency, 77.8% of the women self-reported having never been abused or only once in the last 12 months, whereas 13.1% reported abusive experiences repeating few times, and 9.9% repeating many times. In terms of severity, 66.7% of the older women had not been abused, 22.3% had suffered one type of abuse, and 11.1% two or more. Similarly, 15.0% reported one abusive experience, 8.1% two, and 10.3% three or more abusive events during the last 12 months. Severity measures showed similar associations: social support and high socioeconomic level as protective factors among those with less severe abuse, whereas increased depression, food insecurity and functional impairments were associated with more severe experiences of elder abuse. Frequency followed a different pattern, depressive symptoms were significantly associated with those with few experiences (compared to those with none or once), while functional impairments were associated with many experiences of elder abuse.

**Conclusions:** It is relevant to assess elder abuse through its severity and frequency as inter-individual variability and the complexity of the experience shows different determinants suggesting a syndemic approach. This has important clinical and policy implications.

## Introduction

The World Health Organization (WHO) defines elder abuse as a “single or repeated act, or lack of appropriate action, occurring within any relationship where there is an expectation of trust which causes harm or distress to an older person” (1). Elder abuse is a serious violation to human rights (2) and it includes physical, sexual, and psychological abuse, as well as caregiver neglect and financial exploitation (3). According to a recent meta-analysis, prevalence of elder abuse in community settings is 1 in 6 elder adults worldwide, which accounts for ≈141 million people (2). This is particularly alarming as the literature suggests that elder abuse is associated with increased risk of morbidity and mortality, institutionalization and hospitalization (3), and has negative effects on families and communities (2). Prior research has identified several risk factors for elder abuse, such as shared living situation, dementia, and social isolation (4). In terms of psychosocial resources (i.e., social networks, social support, coping resources), it has been reported that older adults with fewer resources are more vulnerable to abuse and, at the same time, abuse seems to be particularly detrimental to psychological well-being (5, 6).

Elder abuse should not only be seen as a clinical or as a social work problem, it is a public health concern and a public policy issue. In fact, there has been an increasing recognition of elder abuse as a form of family violence which requires a preventive approach (7). However, there are still important gaps in the scientific literature for a full understanding of the phenomenon and evidence-based courses of action. Estimating the magnitude of elder abuse is a first step to formulate policies but discovering the prevalence of elder abuse is inherently difficult, as oftentimes cases are not reported, and victims may not even be aware of what constitutes abuse (7, 8). Despite such difficulties, population-based prevalence studies have advanced our understanding of the scope of elder abuse (2, 9–13). However, severity and frequency of elder abuse is another relevant aspect which has been seldom reported even though the information is readily available in these types of scales. Prevalence studies have used binary outcomes (abused/non-abused) leading to compress the range of the phenomena into one category (14). Elder abuse could be more accurately represented by its severity and frequency, as for many older adults—especially women—abuse is not an isolated event, and they suffer repeated abusive acts of the same type and /or different types of abuse at the same time (14, 15). Integrating severity and frequency variation to elder abuse operationalization can lead to a better grasp of the nature of the phenomena.

Few studies have centered in reporting elder abuse severity and frequency variations, but the available research suggests that repeated and multiple abuses have negative health effects on the older adults, among women possibly even more than when comparing abuse/non-abuse (15). Dong and Simon (3) reported that elder abuse victims who experienced two or more forms of abusive acts had significantly higher rates of hospitalization than those with only one form of abuse. In addition, other studies have reported clear variations in the distribution of severity and frequency of elder abuse subtypes (14–17), but there is still room to better characterize such variations in different settings (i.e., middle-income countries, which are rapidly aging), as well as to identify risk factors linked to higher frequency and severity.

Considering the differential covariation of elder abuse severity and frequency with key psychosocial and sociodemographic predictors also contributes to identifying relevant psychiatric manifestations, such as syndemics [i.e., the aggregation of two or more diseases or health conditions in which some level of deleterious behavior exacerbates the negative health effects of the diseases involved (18)]. The syndemics approach goes beyond the assumption that comorbidities simply occur in tandem to argue that diseases are shaped by local circumstances through especially adverse interactions in contexts characterized by poverty, stress, and structural violence (18). For instance, one of the most investigated syndemics relationships is SAVA—the interaction of substance abuse, violence, and AIDS in individuals living in low-income urban environments, which yields a more harmful combination than any of these conditions in isolation (18) Likewise, diabetes and poverty frequently cluster together, but they interact with depression in countries that vary by income, health system, and cultural values (19). The importance of syndemics is that its interactions amplify disease burdens, reduce the effectiveness of common interventions, and can increase treatment costs (20). If predictors of elder abuse differ according to its severity and frequency, then it is more likely to confound according to different profiles of elder abuse, as the ones revealed by syndemics in other public health issues.

In the Mexican population, there have been prior estimations of elder abuse prevalence. However, prevalence estimates do not always coincide, ranging between 8.1 and 33.4% among non-institutionalized older, but they agree that the most frequent type of abuse is psychological (5, 21, 22). Given the accelerated aging process in middle-income countries like Mexico and the lack of research on both severity and frequency of abuse, as well as its potential syndemic relationships, it is fundamental to add evidence to this body of literature. Such research can lead to better clinical and public policy interventions.

Using population-based data representative of a sample of older women who attend community centers in Mexico City and who were cognitively intact at the time of the interview, in the present cross-sectional research we examine: (1) how elder abuse severity and frequency can be operationalized when using a multiple-item validated elder abuse scale in Mexico; (2) what is the frequency and severity of elder abuse in a sample of urban Mexican older women; and (3) what is the relationship of elder abuse severity and frequency with psychosocial and sociodemographic factors, and whether some syndemic relationships can be highlighted for further research.

## Materials and Methods

### Data Source and Study Population

Data was obtained from a cross-sectional study of older adults who assist to the network of community centers from the Mexican National Institute for Older Adults (INAPAM for its acronym in Spanish). The sampling frame consisted of 113 community centers located in the 16 boroughs (i.e., “Delegaciones”) of Mexico City. These community centers provide a wide array of services that span from health information to leisure activities, such as handcrafts and dancing classes. However, these centers do not provide medical nor mental health services due to limited financial resources. The sample was first stratified by the socioeconomic level of the neighborhood where the community centers are located, and then 36 of them were randomly selected. Between April and December 2014, within the selected centers, all individuals 65 years and older residing in Mexico City were invited to participate on the ENSAAM survey (i.e., the Nutrition and Health Survey of Older Adults, for its acronym in Spanish). Older adults that were not sufficiently functional to answer to the questionnaire unassisted were excluded. The respondents' functionality and cognitive abilities were sufficient to walk by themselves, use public transportation, and participate in the weekly center's activities. With the exception of two members, all the older adults in the centers were able to answer the questionnaire unassisted. The original sample included 576 older adults. Since 92.7% of the participants in the sample were female, males were excluded from the present study; the total sample size was 534 females.

A team of trained interviewers gathered the data in face-to-face interviews. The interviewers read, explained, and answered questions about the informed consent to every older adult. The majority of participants approached agreed to participate (99.4%). This high participation rate was probably achieved due to factors, such as incentives (all participants were offered an individualized nutrition and basic health assessment report), establishing contact with the community group before data collection, and interviewers' training. Data collection took place in the community centers and the mean duration of the interview was 30 min. The research protocol was reviewed and approved by the Research Bioethics Committee of Universidad Iberoamericana (Approval number: 28102013).

Elder abuse was assessed using the Geriatric Mistreatment Scale (GMS), which was developed in Spanish to diagnose elder abuse among Mexican urban elderly (21). The scale has adequate psychometric properties, and it had acceptable internal reliability in this sample (Cronbach's alpha = 0.83). The 22-items of the GMS evaluate the presence of five types of abuse in the last 12 months: physical abuse (5 items), psychological abuse (6 items), care-givers neglect (4 items), financial exploitation (5 items), and sexual abuse (2 items). The response options for each item are dichotomous (i.e., Yes or No).

When using the common scoring of the GMS, one or more positive answers in any of the 22-items signals the presence of abuse and categorizes the older adult as being abused, regardless of the intensity of the behavior. In spite of measuring different types of abuse, the scale is frequently used as a dichotomous outcome. The present study profits from additional information in the scale to estimate the severity and frequency of the abuse. The intensity of elder abuse was operationalized in two ways, as the number of abuses (i.e., number of affirmative responses to any of the 22-items) and as the number of types of abuses. A third dependent variable examines the frequency of each abusive event.

#### Number of Abuses

The total number of affirmative responses to any of the 22-items from the GMS, was recoded and transformed into an ordinal variable with four categories: none, one, two, three, or more abuses. This operationalization treats each abusive event as equal—regardless of its type—and thus allows to measure the intensity by accounting for multiple events.

#### Number of Types of Abuses

The previous positive answers to each event were first classified by type of abuse (i.e., physical, psychological, neglect, financial, or sexual). Sexual abuse had a prevalence of 1.12%, so it was collapsed with physical abuse. Then a new score added the number of types of abuses a person suffered and an ordinal variable with three categories indicated whether the older adult had: none, one, or two or more types of abuse.

#### Frequency of Abuse

The most common operationalization of abuse inquires if an abuse occurred or not. However, a rarely used section of the GMS, examines how often each abusive event happened (i.e., a Yes answer to any of the 22-items). For each positive answer the scales probes if the presumed abusive behavior “never” happened, if it occurred “once,” if it occurred a “few times,” or if it happened “many times.” In the present study, the “once” category was seldom answered so it was collapsed with “never” and an ordinal variable with the three remaining options was used.

#### Depression

Depressive symptoms in older adults were evaluated using the 15-item Geriatric Depression Scale (GDS-15). The scale is widely used in Latin America because, in addition to its psychometric properties, it does not confound physical with depressive symptoms (23). The GDS-15 is a short version of a longer instrument. It was validated in English against clinical criteria with a sensitivity of 92% and a specificity of 89% (24). The GDS-15 was subsequently translated to Spanish, and a content validity examination was performed in Colombia, yielding an appropriate internal reliability with an estimate of 0.73 using the Kuder–Richardson 20 formula (25). The scale's response options are dichotomous and an additive total score was estimated. Using previously established cutoff points in Latin America (25), older adults scoring between 0 and 5 were categorized as non-depressive and those scoring 6 or more were categorized as having depression symptoms.

#### Food Insecurity

We evaluated food insecurity through the Latin American and Caribbean Food Security Scale (ELCSA for its acronym in Spanish), which is a 15-item scale with dichotomous response options that has shown excellent psychometric properties throughout Latin America, including Mexico (26). We used a modified version of the scale adapted for urban Mexican older adults which has also been reported to have good face validity and strong psychometric properties (27, 28). The scale distinguishes between households with children (8 exclusive items) and without them (7 common items). The total score categorizes food secure households and three thresholds of food insecurity: mild, moderate and severe food insecurity, However, given the sample size of present study, the “moderate/severe” food insecurity were collapsed. Considering that both moderate and severe food insecurity imply important challenges to access of nutritious foods, this measurement strategy has been performed in prior studies (29). Therefore, in households without children, 1–3 affirmative answers are categorized as mild food insecurity and 4–8 affirmative answers as moderate/severe food insecurity; likewise, in households with children, the cut-off points are 1–5 for mild food insecurity and 6–15 for moderate/severe food insecurity. A household with zero affirmative answers is considered food secure. Thus, the ordinal variable has three categories indicating the severity of food insecurity.

#### Perceived Social Support

The three-item Oslo-scale (OSS-3) measures the quantity and satisfaction of an individual's perceived social networks (30). The scale has adequate psychometric properties; it has been used in prior studies with older adults (5, 30–32), and it has been reported to have predictive validity with psychological distress (30). The three items were measured as ordinal variables and then added in a total score: higher scores indicated stronger support. The total score was then recoded into three categories: poor (i.e., score between 3 and 8), moderate (i.e., a score between 9 and 11), and strong social support (i.e., a score between 12 and 14).

#### Functionality

Activities of daily living (ADL) and instrumental activities of daily living (IADL) were measured using the Katz ADL (33) and the Lawton IADL scales (34). The Lawton scale measures instrumental activities, such as managing medicines and money, shopping, and making a meal by themselves. Additionally, the Katz scale evaluates non-instrumental abilities, such as bathing, toileting, or walking by themselves. Items from both scales were categorized into a dichotomous variable indicating the presence of a disability in any of the items. Hence, having at least one ADL or one IADL led to a positive dichotomous response.

#### Comorbidities

A continuous summative variable measured the 10 most common comorbidities that older women self-reported had been diagnosed by a health professional. Such comorbidities included diabetes, high-cholesterol, hypertension, heart disease, cancer, asthma, arthritis, osteoporosis, kidney disease, and gastritis. These represent top causes of mortality among older Mexican adults and also the leading causes of hospitalization in this age group (excluding mental health problems) (35). For example, if an older woman only self-reported diabetes, then the variable would be one, whereas an older woman self-reporting a diagnosis of hypertension, cancer and diabetes the variable would be 3.

#### Neighborhood Socioeconomic Status

This variable captured the socioeconomic level of the neighborhood where older adults lived, and it was coded by zip code. The measure comes from Mexico City's index for social development 2010 (36), which used household-level socioeconomic data from the national census to aggregate it at neighborhood level (“*colonia*” in Spanish). It was used as an ordinal variable with three response options: low, medium, and high neighborhood socioeconomic status.

#### Age

It was kept as a continuous variable in years and for some of the analyses was transformed into its quadratic expression to account for non-linear trajectories.

### Statistical Analyses

Descriptive statistics were estimated to portray the prevalence of elder abuse first as a dichotomous measure and then by type of abuse. Due to the distribution of most continuous variables, the median was computed; whereas proportions were estimated for categorical variables. We did Mann-Whitney, ANOVA, and Chi-square tests to compare the characteristics of older women with and without abuse experiences.

Afterwards, we estimated the severity and the frequency of elder abuse, as measured in the three dependent variables discussed above.

Multinomial regression models were estimated separately for each of the three dependent variables. The three parallel models aimed to assess whether predictors have different associations according to the frequency and severity of elder abuse. The reference category for the three models was always the absence of elder abuse. Estimations are presented in odds ratios (OR) to ease the interpretation. All analyses were conducted with STATA 14 (37).

## Results

Table 1 summarized the descriptive statistics of the sample. The sampled population had a median age of 74 years old and 72% of respondents had less than elementary school. The median number of comorbidities was 3. In addition, 52.1% of the sampled older women had at least one disability (i.e., ADL or IADL), and 80.8% of the respondents did not report depressive symptoms. Moreover, 27% reported living alone and 35.3% perceived to have strong social support, 45.9% moderate, and 18.8% poor. According to the estimations performed through the ELCSA, 63.2% of the old women lived in food-secure households 24.5% in mildly food insecure households, and 12.3% in moderately or severely food insecure households. More than half of the older women lived in neighborhoods with low socioeconomic level, around one-quarter (27.8) in medium socioeconomic level (27.8%) and 16.9% in high socioeconomic level neighborhoods.

**Table 1:** Descriptive statistics for the total sample and comparisons between older adults reporting abuse and those who do not report abuse.

	**Total**	**OA with abuse**	**OA without abuse**	**Test**
**Characteristics of participants**	***N*** = **534**	***N*** = **178**	***N*** = **356**	***P-value***
Age, median (IQR)	74 (11)	73 (11)	74 (10)	0.43_a_
**EDUCATION, % (*****N*****)**				
Less than primary	72.20 (187)	78.57 (66)	69.14 (121)	0.26_b_
Primary completed	8.88 (23)	5.95 (5)	10.29 (18)	–
Middle to graduate	18.92 (49)	15.48 (13)	20.57 (36)	–
Household size, median	2 (3)	3 (3)	2 (3)	0.27_a_
Lives alone, % (*n*)	27.05 (142)	26.44 (46)	27.35 (96)	0.82_b_
Number comorbidities, median (IQR)	3 (2)	3 (2)	3 (2)	0.14_a_
**DEPRESSION, % (*****n*****)**				
Risk-depression	19.20 (91)	35.19 (57)	10.90 (34)	$0.00_{\text{b}}^{***}$
**SOCIAL SUPPORT, % (*****N*****)**				
Poor	18.81 (95)	28.92 (48)	13.86 (47)	$0.00_{\text{b}}^{***}$
Moderate	45.94 (232)	46.99 (78)	45.43 (154)	–
Strong	35.25 (178)	24.10 (40)	40.71 (138)	–
**SOCIOECONOMIC STATUS, % (*****n*****)**				
Low	55.29 (282)	68.79 (119)	48.37 (163)	$0.00_{\text{b}}^{***}$
Medium	27.84 (142)	21.97 (38)	30.86 (104)	–
High	16.86 (86)	9.25 (16)	20.77 (70)	–
**FOOD INSECURITY, % (*****n*****)**				
Without FI	63.22 (318)	48.80 (81)	70.33 (237)	$0.00_{\text{b}}^{***}$
Mild	24.45 (123)	30.72 (51)	21.36 (72)	–
Moderate/Severe	12.33 (62)	20.48 (34)	8.31 (28)	–
**FUNCTIONALITY (KATZ), % (*****n*****)**				
At least one	52.06 (278)	62.92 (112)	46.63 (166)	$0.00_{\text{b}}^{***}$

OA, older adults. Significance ^***^ p < 0.01.

a A Mann-Whitney test on the equality of medians was conducted.

b *A Pearson chi-square test was conducted*.

**Figure 1:**
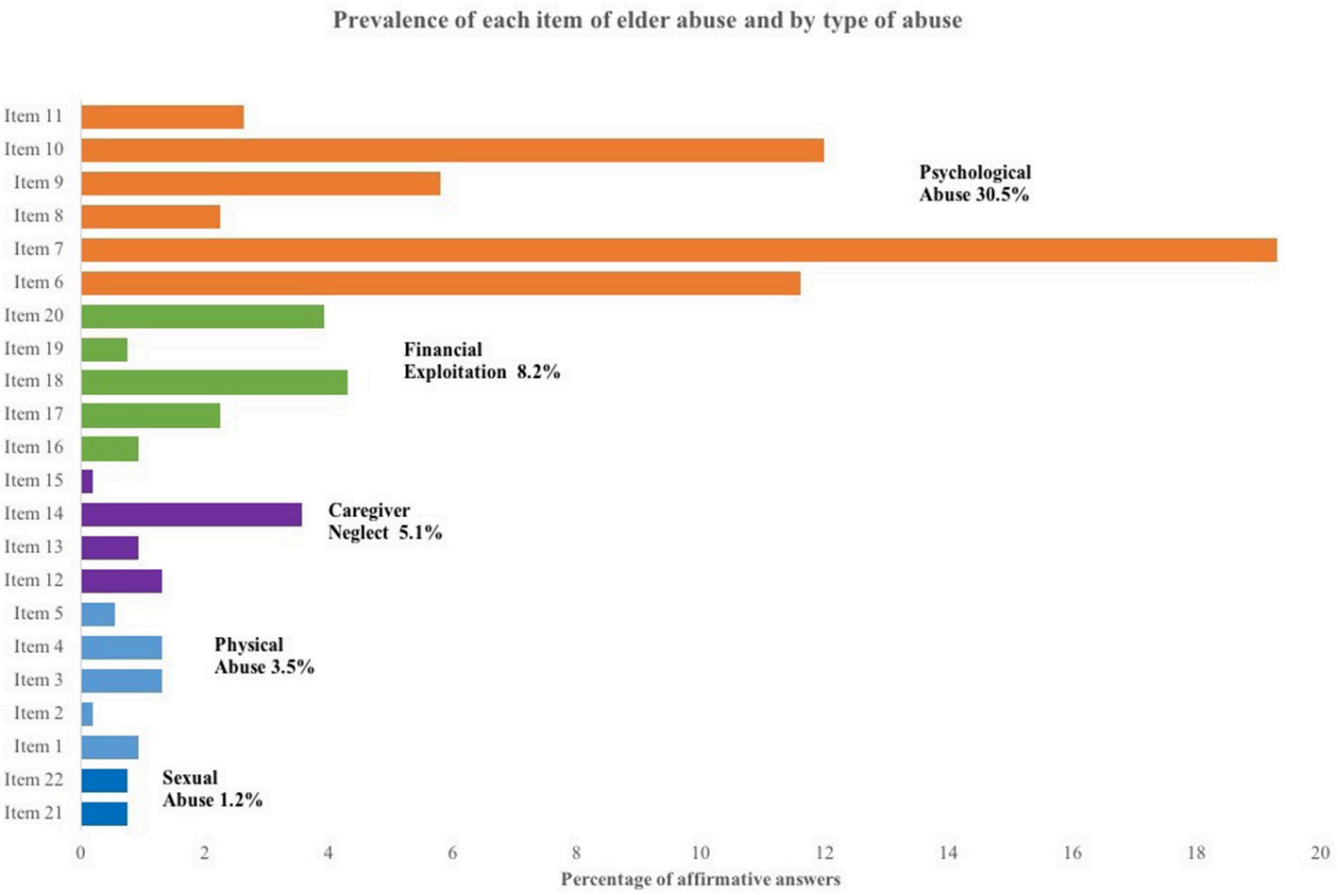
Prevalence of elder abuse by item and by type of abuse. The prevalence of each item is in percentages obtained from a sample of 534 female older adults. Some respondents suffer two or more types of abuse. The items were sorted by prevalence of type of abuse.

With the usual estimation of the GMS, the prevalence of elder abuse in our sample of urban Mexican women was 33.3%. Furthermore, the prevalence by type of abuse was 4.7% for the combination of physical and sexual abuse; 30.5% for psychological abuse; 5.1% for caregiver neglect; and 8.2% for financial exploitation (see Figure 1). Comparisons between abused and non-abused older adults indicate important differences (see Table 1). Older adults reporting abuse had significantly higher rates of depressive symptoms (35.2 vs. 10.9%), and significantly lower social support (28.9 vs. 13.9%). Likewise, adults reporting abuse lived in neighborhoods with a significant lower socio-economic status (68.8 vs. 48.4%) and also had a significantly higher prevalence of moderate/severe food insecurity (20.5 vs. 8.3%). Older women who reported elder abuse also had a higher proportion of functional impairments than their non-abused counterparts (62.92 vs. 46.63%).

**Figure 2:**
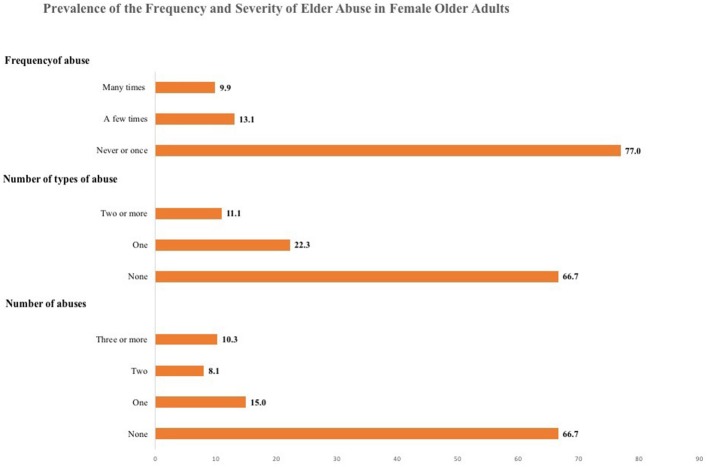
Prevalence of the frequency and severity of elder abuse in Mexican Female Older Adults. The prevalence of each type is percentages obtained from a sample of 534 female older adults.

**Figure 3:**
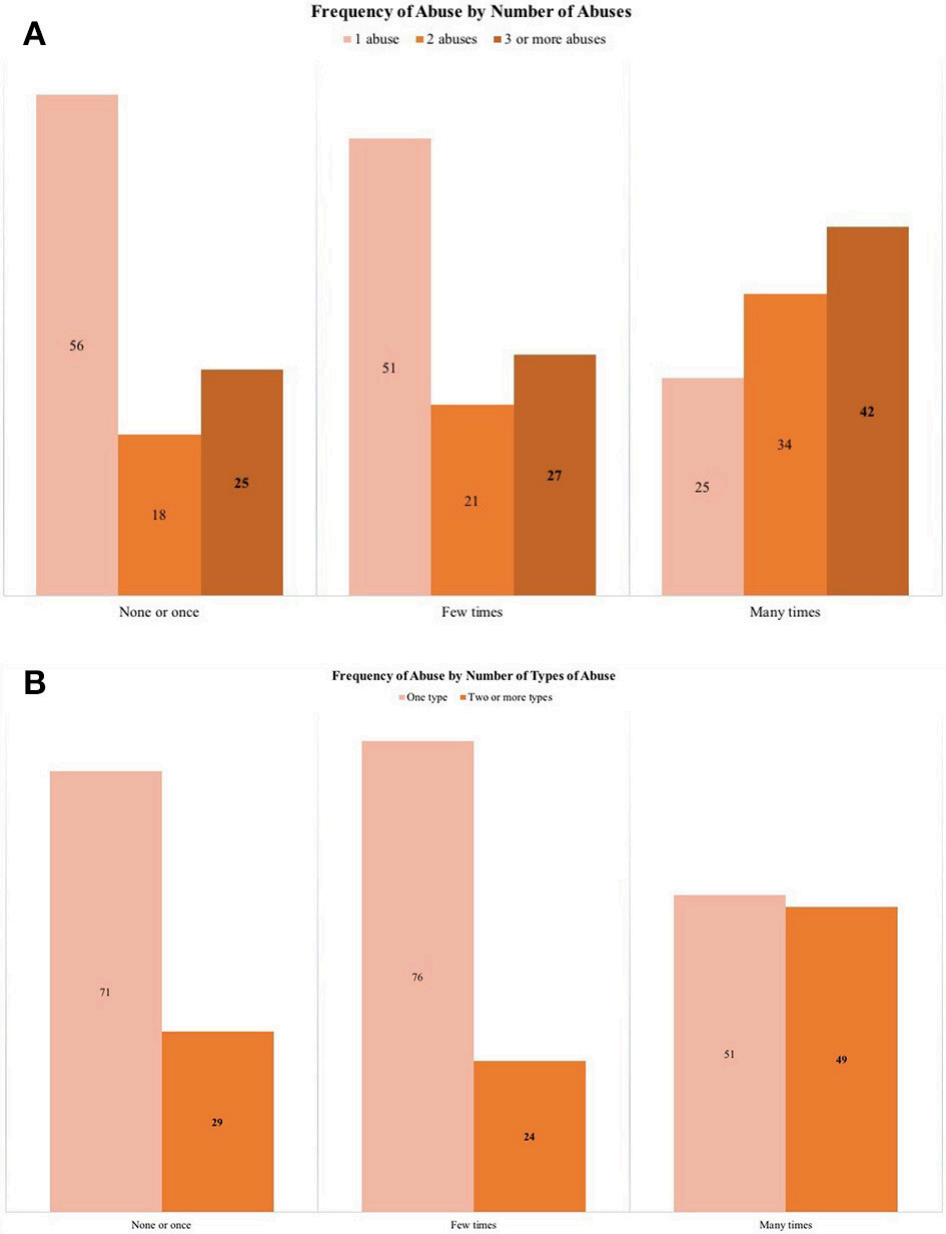
**(A,B)** Associations between frequency and severity of elder abuse. The prevalence is percentages obtained from a sample of 534 female older adults.

A close examination of each item in the GMS revealed considerable variability within types of abuse (see Figure 1), which suggests a more complex phenomenon beyond the dichotomous prevalence. The use of additional information from the scale yielded a more nuanced estimation of the prevalence of the frequency and severity of elder abuse (see Figure 2). The severity of elder abuse was assessed by the number of affirmative answers: 15% reported one, 8.1% two, and 10.3% reported three abuses. The common counting of abuses (as it has generally been operationalized in prior research), would have clustered respondents with one abuse with those with two or more abuse experiences, although it is likely that elder women with one, two or three and more abuses are living different circumstances (14). Similarly, 22.3% experienced only one type of abuse and 11.1% answered with affirmative responses to two or more types of abuse. These different experiences are relevant as prior research has already highlighted that suffering more than one type of abuse leads to different health outcomes (3). The frequency of these behaviors was asked after participants acknowledged the abusive event happened sometime in the last 12 months, and then a perception-based question inquired how frequently the abuse had happened. Seventy-seven percent reported experiencing the abusive event had never occurred or it occurred only once. However, 13.1% established that they had suffered such abuse a “few times” and 9.9% recognized that the abuse occurred “many times.” Importantly, frequency and severity measures of elder abuse are closely associated. Figure 3 shows that older women who reported suffering an abuse “many times” in the last 12 months also report a higher proportion in the number of affirmative responses to abuse and in the number of types of abuses.

Multinomial models specified the associations between key predictors and the three dependent variables of severity and frequency of elder abuse (see Table 2). The first two models assess the predictors for severity. When the number of abuses was the dependent variable, higher socioeconomic status in the neighborhood was associated with reporting one abusive event but not with reporting two or more. The same pattern was observed with social support; when compared with non-abused elders, an affirmative answer was associated with less social support, but the association was not present when the abuse was more severe (i.e., two or more events of abuse). Conversely, depression was not significant for a single affirmative answer when compared with non-abused elders, but it was more likely to occur (OR 2.42) with two abuses and even more likely in the presence of three events of abuse (OR 5.08). A similar pattern was observed for functionality (i.e., ADL or IADL), which was only significant in those elders reporting two or more abusive events (OR 2.36 and 2.18, respectively). Food insecurity was only significantly associated with the most severe levels of elder abuse, i.e., among elder women who reported 3 or more abusive events (OR 1.83). A key result was that predictors differed between women reporting one abuse from those reporting two or more.

**Table 2:** Multinomial models for the severity and the frequency of elder abuse.

**Number of abuses**	**One**		**Two**		**Three or more**	
	**OR**	**CI**	**OR**	**CI**	**OR**	**CI**
Social support	0.59^**^	0.39–0.90	0.71	0.42–1.22	0.74	0.44–1.24
Risk of depression	1.34	0.60–2.99	2.42^*^	0.97–5.99	5.08^***^	2.23–11.57
Food insecurity	1.19	0.76–1.84	1.32	0.76–2.27	1.83^**^	1.12–2.99
Functionality	1.06	0.58–1.94	2.36^**^	1.04–5.33	2.18^**^	1.00–4.75
Comorbidities	1.07	0.88–1.29	1.11	0.88–1.41	0.97	0.77–1.22
N. socioeconomic status	0.55^**^	0.34–0.87	0.81	0.47–1.41	0.95	0.56–1.61
Age^2^	1.00	1.00–1.00	1.00	1.00–1.00	1.00	1.00–1.00
Constant	0.41	0.03–4.37	0.05	0.00–1.21	0.02	0.00–0.56
**Number of types abuses**	**One**		**Two or more**			
	**OR**	**CI**	**OR**	**CI**		
Social support	0.68^**^	0.47–0.98	0.61^*^	0.37–1.02		
Risk of depression	2.02^**^	1.04–3.89	3.72^***^	1.61–8.57		
Food insecurity	1.19	0.81–1.73	1.92^***^	1.17–3.13		
Functionality	1.14	0.68–1.91	3.62^***^	1.59–8.25		
Comorbidities	1.12	0.96–1.32	0.89	0.70–1.13		
N. Socioeconomic status	0.56^***^	0.38–0.83	1.14	0.69–1.90		
Age^2^	1.00	1.00–1.00	1.00	1.00–1.00		
Constant	0.32	0.04–2.46	0.03	0.00–0.67		
**Frequency of abuse**	**Few times**		**Many times**			
	**OR**	**CI**	**OR**	**CI**		
Social support	0.88	0.56–1.38	0.86	0.53–1.40		
Risk of depression	4.52^***^	2.17–9.40	2.24^*^	0.98–5.13		
Food insecurity	1.11	0.77–1.75	1.35	0.84–2.16		
Functionality	1.75^*^	0.90–3.40	2.12^**^	1.03–4.37		
Comorbidities	0.97	0.80–1.19	1.06	0.86–1.32		
N. Socioeconomic status	0.72	0.45–1.16	0.69	0.41–1.18		
Age^2^	1.00	1.00–1.00	1.00	1.00–1.00		
Constant	0.04	0.00–0.54	0.22	0.01–4.17		

Level of significance:

* p < 0.1;

** p < 0.05;

*** *p < 0.01*.

The associations with the second dependent variable, number of types of abuses, also assessing severity of abuse, shared similar characteristics as the previous one. For elders suffering one type of abuse, the protective factors were higher social support (OR 0.68) and higher neighborhood socioeconomic status (OR 0.56), whereas the most important risk factor was depression. Reporting depressive symptoms was associated with a higher likelihood of reporting one type of abuse (OR 2.02). On the other hand, for elders reporting two types of abuse, social support was a weaker buffer (only significant at *p* < 0.1) and socioeconomic status ceased to be a protective factor. Moreover, depression increased the strength of its association (OR 3.72). Similarly, food insecurity and functionality issues became relevant predictors, both increasing the risk of suffering two types of abuse (OR 1.92 and 3.62, respectively). As in the previous model, the predictors of elder abuse differed according to its severity.

The estimation of the multinomial model when using frequency of abuse as a dependent variable was different from the model using severity of abuse variables. Depression remained a key predictor of elder abuse; there was a strong association among older women reporting that the abusive experiences had happened a few times (OR of 4.52), while the strength of the association was weaker in those reporting the that the abuse had happened many times (OR 2.24 and only significant at *p* < 0.1). The only predictor that became more significant as elder abuse became more frequent was functionality; among older women reporting that the abuse had happened few time, the OR was 175 (only significant at *p* < 0.1) but among those reporting that the abuse had happened many times the OR was 2.12 (and significant at *p* < 0.05). In this model, social support was not a protective factor and food insecurity was not a risk factor as it was observed in the severity of abuse models. Such differences may actually arise from the fact that the frequency measure is based on experience perception.

## Discussion

Elder abuse is a violation to human rights and a public health concern affecting 1 in 6 older adults worldwide (2). Mexico is no exception and previous samples indicate a significantly higher prevalence of 32% in adults 60 years and older (21). Our study focused on females in Mexico City and found a similar prevalence of 33%. As in previous studies, psychological abuse was the most common with nearly 30% reporting this type. However, a closer analysis of the data showed the need for a more nuanced understanding of a complex phenomenon. For instance, item 7 in the GMS scale—“Have you ever been treated with indifference or being ignored?”—is the most frequently identified psychological abuse (19%, in Figure 3) and a single affirmative answer suffices to categorize an older adult with abuse. The common operationalization of the scale misses how such indifference may be worsened first by other forms of psychological abuse, as humiliation (Item 6) or disrespect toward decisions (Item 10), and then by its combination with some form of financial exploitation or caregiver's neglect. Moreover, the common scoring equals events occurring only once with those happening several times. Since the common scoring of elder abuse is blind toward levels of severity and frequency, the study proposed three dependent variables to offer a new perspective on elder abuse.

Elder abuse is defined as a violent event occurring in a relationship with an expectation of trust causing harm and distress. Therefore, the intensity and periodicity of these events are relevant dimensions to assess the extent that disease burden affects an older adult. Our results indicated that the layers of severity are of considerable magnitude and should not be overlooked by the dichotomous estimates of abuse; 8% of the older adults suffer two abuses and 10% three or more, while 11% experienced two or more types of abuse. Therefore, the experience of abuse was not limited to a single expression (i.e., psychological or financial exploitation) but sometimes reflected at least two (i.e., psychological and financial exploitation). Additionally, almost a fourth of the sample recognized repeated abuse; 13% said it occurred a few times and 10% that it happened many times. While overall prevalence did not find gender differences (2), previous research identified that women were more vulnerable to repeated and more intense forms of abuse (15) so these measures may help identify different impacts of abuse by gender (3).

A notable finding in our study is how these two dimensions interrelate. Descriptive statistics show that, as the abuse becomes more frequent, it also becomes more severe. Therefore, higher degrees of elder abuse suggest that the level of distress caused to older adults is probably higher in what may be different experiences of the phenomenon. Therefore, it was relevant to examine if differences in severity and frequency of abuse were also associated with different predictors, especially those related to poverty settings, as food insecurity.

Our models revealed that the protective factors for the less severe degrees of abuse in the two dependent variables were social support and a higher socioeconomic status in the neighborhood. However, these factors ceased to be significant as the severity of abuse increased. Instead, risk factors that were not associated with the less severe degrees of abuse now became significant. Depressive symptoms, food insecurity, and functionality were associated only with the more severe degrees of elder abuse. Importantly, the more severe expressions of abuse revealed unexplored risk factors, such as food insecurity, which is seldom reported in the abuse literature, most likely because it is obscured by dichotomous measures. These findings were similar using two dependent variables operationalized in different ways, thus strengthening the singular clustering of risk factors.

These differential associations by severity suggest two profiles of abuse in which risk and protective factors cannot be assumed to be the same. The most severe profile of abuse indicated a singular clustering of risk factors akin to syndemics. The combination of elder abuse in urban women with depressive symptoms, at least one disability, and reporting food insecurity is likely to interact by amplifying disease burden and reducing the effect of common protective factors, therefore yielding worse distress to the older adult. The clinical significance of syndemics is that the severe manifestation of elder abuse could reduce the effectiveness of usual interventions to mitigate it (20). A worrisome finding for this cluster was that social support is not as influential as a protective factor as it has been reported in prior literature that uses dichotomous versions of elder abuse scales.

Thus, in addition to inquiring on the severity of elder abuse, clinicians may need to screen for predictors, such as risk of depression, functionality, and food insecurity in order to identify these intractable profiles of abuse and design specific interventions for them (7). Moreover, community centers need tailored policies and carefully trained personnel to address the health conditions and thresholds of disease burden to aid addressing different profiles of elder abuse.

Frequency was not a dimension that suggested a different profile of elder abuse. The periodicity of the abuse had stable risk factors. Frequency increased only as a function of the number of disabilities. Depressive symptoms were more strongly associated with occasional abuse rather than with the more frequent abuse, thus it was an inconsistent risk factor. The other risk factors clustering with severity were not significantly associated with the frequency measure, so this vector of the scale was less useful probably because this is a perception based measurement that could require some psychometric adaptations. Unexpectedly, in the multinomial models, as well as in the bivariate statistics, the category of “few times” had a higher prevalence of risk factors than the “many times” category. A likely explanation is the small sample size for the “many times” category. These non-linear associations suggest that recall bias could make it a difficult task to adequately differentiate the periodicity of the abuse in a 1-year timeframe and perhaps fewer categories may suffice. Additional psychometric analyses are warranted in order to identify adequate cutoff points for the measure of frequency of abuse.

The present study had a few limitations. First, the cross-sectional design hampered the specification of the direction of the association between elder abuse and the independent variables, so the descriptive analyses were limited to show the clustering between them. Second, the inclusion criteria to the sample was participation at an INAPAM community center. This meant that older adults who were not able to attend or not willing to do so were not considered in our estimates and thus the sample is biased toward potentially healthier and more functional older adults—most likely underestimating elder abuse. Third, our sample was unable to capture enough males to make significant comparisons, so its results are constrained to females only. Lastly, the small sample size reduced the statistical power to identify significant associations at the more severe end of elder abuse, so several variables had to be collapsed, reducing its variability, and few sociodemographic adjustments were made when estimating the multinomial models.

## Conclusions

This study goes further than presenting traditional prevalence estimates of elder abuse based on scores that reduce the phenomenon to a dichotomous variable (i.e., abuse/non-abuse). In order to understand the inter-individual variability and complexity of elder abuse we proposed different ways to examining severity of elder abuse (number of abuses and number of types of abuse) and frequency (few times or many times) of elder abuse. We were able to identify how different risk factors predicted the most severe types of abuse—although not the most frequent. Using a syndemics approach, these findings helped identify which deleterious risk factors tend to cluster in urban older women living in neighborhoods with low socioeconomic status: symptoms of depression, food insecurity, and at least one functionality impairment (either ADL or IADL). This cluster has clinical and policy significance because a well-known protective factor, such as social support was not associated with the most severe expressions of abuse and the deleterious clustering appeared regardless of the socioeconomic status of the neighborhood. Therefore, this cluster of risk factors may prove to be resistant to common treatments. We expect that our findings will help to improve strategies for prevention, identification, and treatment of the most severe cases of elder abuse.

## Author Contributions

MV-C conceptualized the research question and the hypothesis, and defined statistical models jointly with her co-author. PG-R estimated the statistical models. Both authors discussed the outcomes of the models and their implications, defined the content of the different sections of the paper and jointly wrote it.

### Conflict of Interest Statement

The authors declare that the research was conducted in the absence of any commercial or financial relationships that could be construed as a potential conflict of interest.
